# The systematics of the worldwide endoparasite family Apodanthaceae (Cucurbitales), with a key, a map, and color photos of most species

**DOI:** 10.3897/phytokeys.36.7385

**Published:** 2014-04-30

**Authors:** Sidonie Bellot, Susanne S. Renner

**Affiliations:** 1 Systematic Botany and Mycology, University of Munich (LMU), Menzinger Strasse 67, 80638 Munich, Germany

**Keywords:** Apodanthaceae, genus circumscriptions, mitochondrial DNA sequences, nuclear DNA sequences, parasitic plants, species circumscriptions

## Abstract

Using morphological, nuclear, and mitochondrial data, we here revise the taxonomy of Apodanthaceae and allocate the 36 names published in the family to ten biological species in two genera, *Apodanthes* and *Pilostyles*. All species are endo-parasites that live permanently inside trees or shrubs of the families Salicaceae or Fabaceae and that only emerge to flower. Because of this life history, Apodanthaceae are among the least known families of flowering plants. Nevertheless, the World’s herbaria as of 2013 hold at least 785 collections that, in combination with DNA phylogenies, permit well-founded species circumscriptions and geographic range maps. We also provide a key to all species, discuss the newly accepted or synonymized names, and make available color photos of six of the ten species.

## Introduction

Apodanthaceae Tiegh. ex Takht. (Cucurbitales) is a family of endoparasites that live entirely in their host’s stems and only become visible once the strictly unisexual flowers have burst through the bark. This life style, added to the small size of the flowers and patchy occurrence of the apparently mostly dioecious populations, has made it difficult to collect good and complete herbarium material (including both sexes and flowering and fruiting specimens). While populations once identified may be recollected at the same time year after year, usually only local botanists will have the opportunity to carry out such recollections. Apodanthaceae are disjunctly distributed in North and South America, mainland Africa, Iran, and Australia. They occur in arid as well as humid tropical environments. Two genera have been validly described, the worldwide *Pilostyles*, and *Apodanthes* from Central and South America.

The taxonomy of the genera and species of Apodanthaceae has not been studied since the work of Ida de Vattimo-Gil (Vattimo-Gil 1950, 1955, 1970, 1973). Modern molecular-phylogenetic work based on representatives of most of the so-far named species (Bellot and Renner in review), together with study of collections deposited in the World’s herbaria since the end of the 19th century, has revealed the need to synonymize many superfluous names, a task that we carry out here. We also up-date the circumscription of the family and its two genera, and clarify that they have specialized on different hosts, namely Salicaceae (mainly *Casearia*) and Fabaceae.

To achieve a better understanding of species boundaries and relationships, and to clarify the species’ geographic and host ranges, we compared loans from numerous herbaria, dissected flowers, and isolated DNA from multiple collections. Molecular markers useful for these obligatory holoparasites are the nuclear 18S ribosomal RNA region and mitochondrial *matR* (Barkman et al. 2004; Bellot and Renner in review), and we show here that these markers can be used to place incomplete collections (for example, those of only one sex or only with fruits) in the correct species. Lastly, we provide an annotated key to all species that we recognize, and brief descriptions of their diagnostic traits along with color images and comments on their geographic and host ranges.

## Methods

### Plant material, DNA extraction and sequencing, phylogenetic analyses

We enlarged the DNA data matrix of Bellot and Renner (in review) by extracting DNA from additional specimens representing either unusual individuals or potential new species. No DNA sequences could be obtained from *Pilostyles stawiarskii*, known only from two collections in R, and *Pilostyles holtzii*, the only collection of which was destroyed in World War II. Suppl. material 1 shows species names and their authors, herbarium vouchers, and GenBank accession numbers. In total, 10 sequences (3 of 18S and 7 of *matR*) were newly generated for this study.

Total genomic DNA was extracted from herbarium specimens using the commercial plant DNA extraction Invisorb® Spin Plant Mini Kit (Stratec molecular, Berlin, Germany). The mitochondrial *matR* and the nuclear 18S genes were amplified using the primers listed in Bellot and Renner (in review). PCR products were purified with the ExoSAP or FastAP clean-up kits (Fermentas Life Sciences, St. Leon-Rot, Germany), and sequencing relied on the Big Dye Terminator v. 3.1 cycle sequencing kit (Applied Biosystems, Foster City, CA, USA) and an ABI 3130-4 automated capillary sequencer. Chromatograms were checked and sequences were edited using Geneious R7 (Biomatters, available from http://www.geneious.com), and contigs were then blasted against GenBank to rule out contamination. Alignments of the clean sequences were performed using the program MAFFT v. 7 (Katoh 2013) resulting in matrices of 1626 and 1727 aligned nucleotides for *matR* and 18S, respectively. We failed to amplify the gene *matR* from the African *Pilostyles aethiopica* and from the Iranian *Pilostyles haussknechtii*. Phylogenetic reconstructions relied on maximum likelihood (ML) as implemented in RAxML-7.2.8-ALPHA (Stamatakis 2006), using the GTR + G model of nucleotide substitution with 100 bootstrap replicates under the same model. Trees were rooted on *Corynocarpus laevigatus* (Corynocarpaceae; Cucurbitales), based on Filipowicz and Renner (2010).

### Morphological data and assessment of the host ranges of Apodanthaceae

We geo-referenced locality data from 785 herbarium collections on loan from the herbaria B, G, C, GH, K, M, MO, MSB, W, NA, PMA, and SI and added data from the Global Biodiversity Information Facility (GBIF Backbone Taxonomy, 2013-07-01, http://www.gbif.org/species/7279680). We also recorded host names, up-dating their taxonomy as relevant. All label information was compiled in a database using the Botanical Research and Herbarium Management System (BRAHMS, http://herbaria.plants.ox.ac.uk/), and maps were produced using DIVA-GIS 7.5 (http://www.diva-gis.org). Collections were sorted by geography, flowering specimens were sexed to evaluate sexual dimorphism, and a representative number of flowers were then dissected under a stereoscope. For each dissected flower, the first author recorded the number, arrangement and size of the tepals, shape and ornament of the pistil/central column, number of pollen sacs, presence of hairs and presence of a nectary at the base of the flower. Pictures of representative organs were taken using a Dino-Lite USB microscope model AM413ZT (Dino-Lite Europe) and the DinoCapture Imaging software version 2.0 of the same company.

## Results and discussion

### Genus and species boundaries in Apodanthaceae

The dissections showed that species have characteristic flower sizes, number of tepals, tepal cilia, and number of anthers rings. For the American species, we use these differences in the key (below). Suppl. material 2 shows measurements and counts from the 123 dissected flowers. Six collections could not reliably be assigned to these groups because their flowers were slightly unusual: *R. Callejas et al. 8062*, a male plant from Colombia identified as *Apodanthes caseariae* by A. Idarraga in 2002; *Y. Mexia 4540*, a female plant from Brazil that is the type of the name *Apodanthes minarum*; *H. S. Irwin et al. 20350*, a female plant from Brazil identified as *Pilostyles ulei* by Ida de Vattimo in 1975; *H.S. Irwin 31560*, a male plant identified as *Pilostyles blanchetii* by the first author but parasitizing an uncommon host (*Dioclea*, Fabaceae); *J. Rzedowski 11303*, a female plant from Mexico identified by the collector as *Pilostyles thurberi*; and *F. Chiang 9034*, a female plant from Mexico identified as *Pilostyles thurberi* by J. Henrickson in 1972.

**Figure 1.**
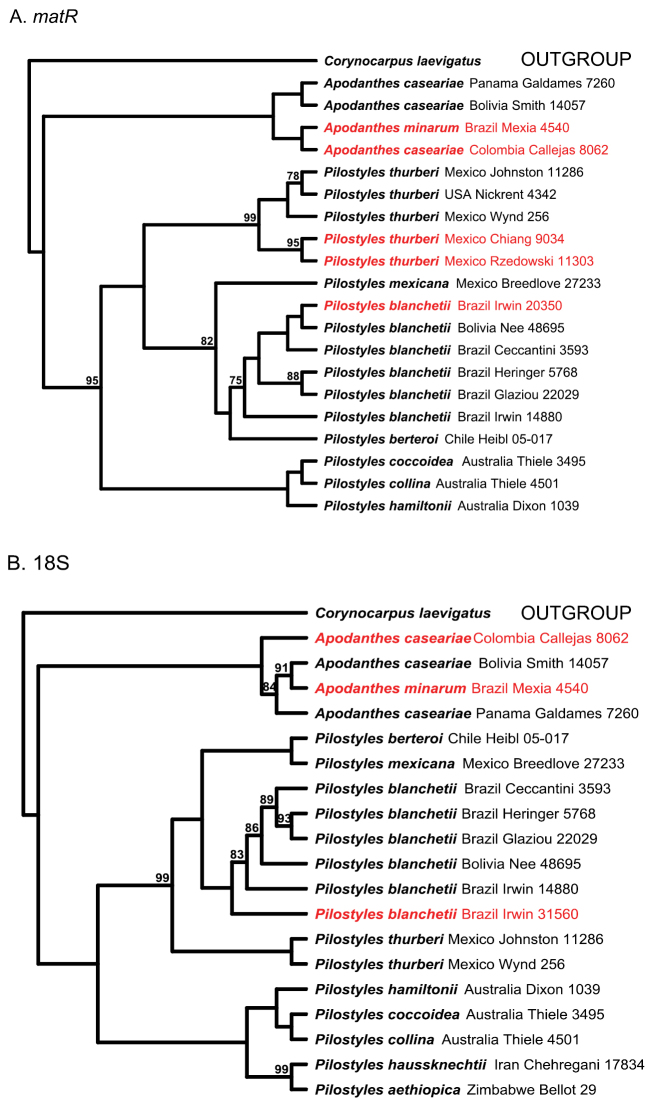
Phylogenetic relationships in Apodanthaceae obtained under maximum likelihood from the mitochondrial gene *matR* (**A**) and the nuclear ribosomal RNA gene 18S (**B**). Shown in red are the specimens we wanted to identify to species. Numbers indicate bootstrap support >70%.

The 18S and *matR* molecular trees show the *Pilostyles* collections that we wanted to identify (in red on Fig. 1) grouped with *Pilostyles thurberi* or *Pilostyles blanchetii*. The collections *R. Callejas et al. 8062* and *Y. Mexia 4540* grouped with two undoubted representatives of *Apodanthes caseariae*. *R. Callejas et al. 8062* is a male plant and comes from the border with Panama, a country where *Apodanthes caseariae* has been repeatedly collected. The host of *R. Callejas et al. 8062* was originally identified as *Trema* (Cannabaceae), but a partial *matR* sequence of this host BLASTed to *Casearia nitida*, making it likely that the host was in fact a *Casearia*. If that is the case, this would suggest that the collection represents an *Apodanthes*. The few male flowers of *Apodanthes caseariae* that have so far been dissected (Suppl. material 2) do not allow assessing the full morphological variability of the male flowers of this species. Therefore we had to rely on DNA for identification. In terms of its *matR* (Fig. 1A) *R. Callejas et al. 8062* was embedded among other sequences of *Apodanthes caseariae*, while in terms of its 18S (Fig. 1B), it was sister to them. We identified the specimen as *Apodanthes caseariae*. Other *matR* and 18S sequences in the *Apodanthes caseariae* clade are from the type of the name *Apodanthes minarum* (*Mexia 4540*) from Brazil. Its host was a *Casearia* and its (female) flowers match those of *Apodanthes caseariae* (Suppl. material 2). We therefore synonymize *Apodanthes minarum* under *Apodanthes caseariae* (an action carried out below).

**Figure 2.**
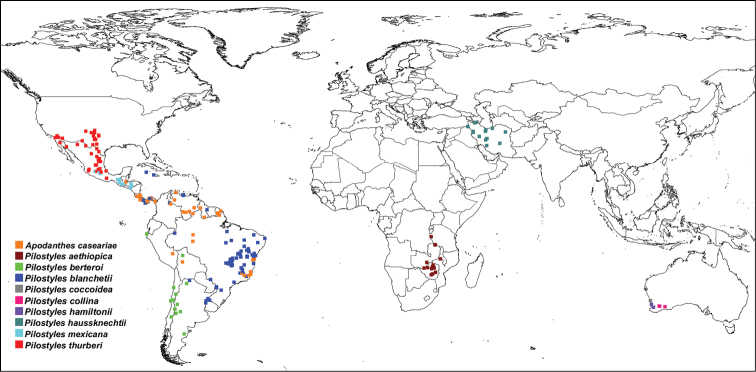
Geographic distribution of Apodanthaceae based on label information from 785 herbarium collections.

**Figure 3.**
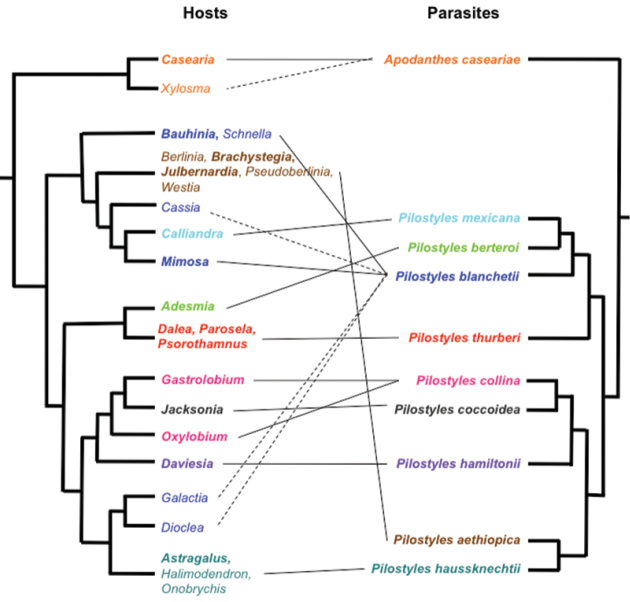
Phylogenetic relationships among the hosts of Apodanthaceae (legume relationships from Wojciechowski et al. 2006) and among the species of Apodanthaceae (from Bellot and Renner in review). Identical colors link parasite species and their host(s) and are also used in Figure 2. Dashed lines represent associations with rarely reported hosts; hosts in bold are the most common ones.

In combination, the present morphological and molecular results show that Apodanthaceae comprise at least ten biological species that can be allocated to two mutually monophyletic genera. In the Americas, these are *Pilostyles thurberi* in the southern United States of America and Mexico, *Pilostyles mexicana* in Mexico, Guatemala and Honduras, the widely distributed *Pilostyles blanchetii* from Panama to Jamaica to Brazil and Uruguay, and *Pilostyles berteroi* in Chile and Argentina. The Americas also harbor *Apodanthes caseariae* from Guatemala to Brazil (Fig. 2). Australia has three species, *Pilostyles coccoidea*, *Pilostyles collina*, and *Pilostyles hamiltonii*; Iran has *Pilostyles haussknechtii*, and Africa has *Pilostyles aethiopica*. The second African species, *Pilostyles holtzii* has not been recollected since 1907, when its type collection was made. Another species, the southern Brazilian *Pilostyles stawiarskii*, is only known from two specimens (one of them the type) collected at the same locality in Jan./Feb. 1948 and Dec. 1949; morphologically it resembles *Pilostyles blanchetii* (Vattimo, 1950). The host ranges of our accepted genera and species do not overlap. *Apodanthes* parasitizes only Salicaceae, whereas *Pilostyles* parasitizes only Fabaceae. As seen on Figure 3, there is a correspondence, although not perfect, between the phylogenies of host genera and parasitic species, and host specialization may have played a role in speciation of Apodanthaceae. At the species level, Table 1 shows that species of Apodanthaceae can grow on one or up to thirteen host species. As seen in Figures 2 and 3, our species concepts are corroborated by geographic and host ranges, except in the case of *Apodanthes caseariae* and *Pilostyles blanchetii*, both widespread in Brazil. These two species have different sized flowers (see below), and parasitize phylogenetically distantly related hosts (Fig. 3).

**Table 1.** Hosts of Apodanthaceae based on label information from 785 herbarium collections. Upper case numbers refer to the references below the table.

**Parasite**	**Host genera**	**Host species**
*Pilostyles aethiopica*	*Berlinia*^1^, *Brachystegia*, *Julbernardia*, *Pseudoberlinia*^1^, *Westia*^1^	*Brachystegia boehmii* Taub., *Brachystegia glaucescens* x *spiciformis*, *Brachystegia spiciformis* Benth., *Brachystegia taxifolia* Harms., *Julbernardia globiflora* (Benth.) Troupin
*Pilostyles berteroi*	*Adesmia*	*Adesmia arborea* Bert. ex Savi, *Adesmia* aff. *spinosissima* Meyen, *Adesmia obovata* Clos, *Adesmia bedwellii* Skottsb., *Adesmia miraflorensis* Remy, *Adesmia uspallatensis* Gill ex H. & A., *Adesmia gracilis* Meyen ex Vogel, *Adesmia microphylla* H. & A., *Adesmia monosperma* Clos, *Adesmia pinifolia* Gillies, *Adesmia trijuga* Gillies
*Pilostyles blanchetii*	*Bauhinia*, *Cassia*, *Dioclea*, *Galactia*^2^, *Mimosa*, *Schnella*	*Bauhinia candicans* Benth., *Bauhinia divaricata* L., *Mimosa claussenii* Benth., *Mimosa cyclophylla* Taub., *Mimosa* aff. *setosa* Benth., *Mimosa maguirei* Barneby, *Mimosa scabrella* Benth., *Mimosa setosissima* Taub., *Mimosa uraguensis* H. & A., *Mimosa* cf. *xanthocentra* Martius, *Schnella cumanensis* Britton & Rose
*Apodanthes caseariae*	*Casearia*, *Xylosma*	*Casearia aculeate* Jacq., *Casearia arborea* Urb., *Casearia decandra* Jacq., *Casearia grandiflora* Cambessèdes, *Casearia guianensis* Urb., *Casearia hirsute* Swartz, *Casearia nitida* Jacq.
*Pilostyles coccoidea*	*Jacksonia*	
*Pilostyles collina*	*Gastrolobium*, *Oxylobium*	*Gastrolobium euryphyllum* Chandler & Crisp
*Pilostyles hamiltonii*	*Daviesia*	*Daviesia angulata* Benth., *Daviesia decurrens* Meissner, *Daviesia pectinata* Meissner, *Daviesia preissii* Lindley
*Pilostyles haussknechtii*	*Astragalus*, *Halimodendron*, *Onobrychis*	*Astragalus brachycalyx* Fisch., *Astragalus brachystachys* DC., *Astragalus cephalanthus* DC., *Astragalus chalaranthus* Boiss. & Hausskn., *Astragalus compactus* Reiche, *Astragalus floccosus* Boiss., *Astragalus gossypinus* Fisch., *Astragalus microcephalus* Willd., *Astragalus rhodosemius* Boiss. & Hausskn., *Astragalus spinosus* Muschler, *Astragalus susianus* Boiss., *Astragalus verus* Olivier, *Halimodendron halodendron* (Pall.) Druce
*Pilostyles mexicana*	*Calliandra*	*Calliandra houstoniana* (Miller) Standley
*Pilostyles thurberi*	*Dalea*, *Psorothamnus*, *Parosela*^3^	*Dalea bicolor* Humb. & Bompl. in Willd., *Dalea formosa* Torrey, *Dalea frutescens* Gray, *Psorothamnus emoryi* (Gray) Rydberg

^1^Verdcourt, B., 1998. Flora of tropical East Africa - Rafflesiaceae. Flora of tropical East Africa 175, 1–2. CRC Press. ^2^Ule, E., 1915. Rafflesiaceae. Notizblatt des Königl. botanischen Gartens und Museums zu Berlin-Dahlem 6, 292–293. ^3^Rose, J. N., 1909. Studies of Mexican and Central American Plants n°6. Contributions from the United States National Herbarium 7, 26–265.

## Description of the family

**Figure 4.**
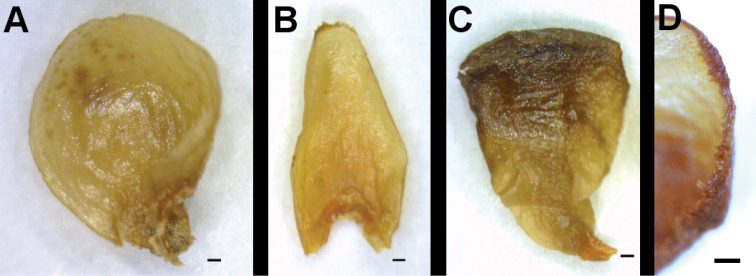
Tepals of Apodanthaceae. **A** Tepal of the outer whorl of *Apodanthes caseariae* **B** Tepal of the middle whorl of *Apodanthes caseariae* **C** Tepal of the inner whorl of *Apodanthes caseariae* **D** Tepal margin of *Apodanthes caseariae*. The scale bar corresponds to 0.2 mm.

**Figure 5.**
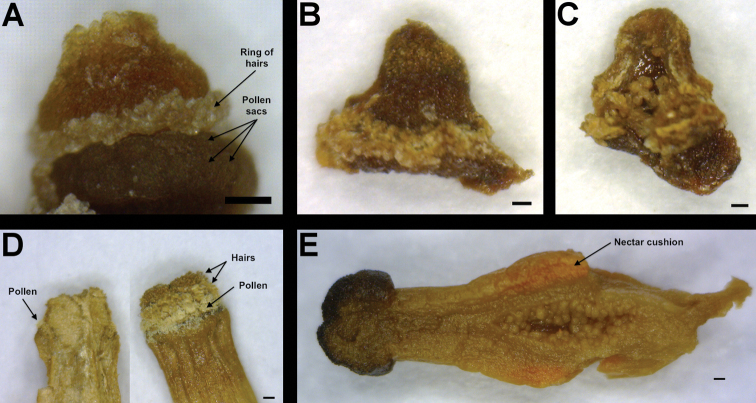
Sexual organs of Apodanthaceae from rehydrated herbarium material. **A** Androecium of *Pilostyles haussknechtii*, note the two rings of pollen sacs topped by a ring of hairs **B** Style and stigma of *Pilostyles haussknechtii* **C** Ovary locule and ovules of *Pilostyles haussknechtii* **D** Androecium of *Apodanthes caseariae* after bursting of the pollen sacs, note the hairs covering the column apex **E** Gynoecium of *Apodanthes caseariae*. The scale bar corresponds to 0.2 mm.

Stem-endoparasites, non-photosynthetic. No leaves, stem or roots, instead an endophytic system of cells inside the stem parenchyma of the host, flowers bursting through the host bark. Flowers unisexual, plants dioecious or monoecious, a point still insufficiently known; flowers of both sexes on the same host or not. Pollination by flies and bees, possibly also wasps (Bellot and Renner 2013; Sipes et al. 2014), based on the fruit color and size, dispersal is probably by birds. Flowers white or yellow (*Apodanthes*), or white, pink, orange, red, purple or brown (*Pilostyles*), aggregated on the host stems, minute (1.5 to 15 mm long when dried), usually with radial symmetry. Perianth composed of 2 or 3, rarely 4, whorls of tepals (Fig. 4A–C), the latter sometimes with hairs along their margins (Fig. 4D), or a hair cushion at their basis (Blarer et al. 2004). In male flowers, the staminal filaments completely fused and forming a tube around a central column that is usually fused to the column (Fig. 5A, D), the up to 72 pollen sacs arranged in 1–4 rings around the column apex (Fig. 5A), the column apex dome-shaped and circled or covered by single-celled hairs (Fig. 5A, D), a basal nectar cushion in both sexes (Fig. 5E). Female flowers without staminodes and with a single thick style topped by the dome-shaped stigma (Fig. 5B, C, E). Ovary semi-inferior, placentation parietal with 50–300 ovules (Fig. 5C, E). Seeds ca. 0.5 mm long (Bouman and Meijer 1994). Fruit a fleshy berry.

### Key to the genera and species of Apodanthaceae

**Table:** 

1a	From the Neotropics, parasitizing Salicaceae, tepals always in 3 whorls with (from the outside) 2, 4, and 4 tepals, the inner whorl easily deciduous, female flowers >5 mm long	1. *Apodanthes caseariae*
1b	From the Neotropics, Africa, Iran, or Australia, parasitizing Fabaceae, number of outer tepals usually >2, female flowers <5 mm long	2
2a	Occurring in Australia	3
2b	Not in Australia	5
3a	Tepals in 3 whorls	2. *Pilostyles collina*
3b	Tepals in 2 whorls	4
4a	Flower diameter >3 mm	3. *Pilostyles hamiltonii*
4b	Flower diameter <3 mm	4. *Pilostyles coccoidea*
5a	Occurring in Africa	5. *Pilostyles aethiopica*
5b	Not in Africa	6
6a	Occurring in Iran	6. *Pilostyles haussknechtii*
6b	Occurring in the America	7
7a	Tepals in 3 whorls, each with 2 to 7 tepals, anthers in 4 whorls (spiral), number of anther lobes >70, on *Adesmia*	7. *Pilostyles berteroi*
7b	Tepals in 3 whorls, each with 3 or 4 (rarely more) tepals, anthers in 2 or 3 whorls, number of anther lobes <70, not on *Adesmia*	8
8a	Middle tepals ovoid, on *Calliandra*, *Dalea*, *Parosela* or *Psorothamnus*, anthers in 3 whorls (at least if on *Dalea*, *Parosela* or *Psorothamnus*)	9
8b	Middle tepals mostly diamond-shaped, apparently never on *Calliandra*, *Dalea*, *Parosela* or *Psorothamnus*, anthers in 2 whorls (females of the three species cannot be securely distinguished)	8. *Pilostyles blanchetii*
9a	On *Calliandra*, tepals in 3 whorls, each with 4 tepals	9. *Pilostyles mexicana*
9b	On *Dalea*, *Parosela*, or *Psorothamnus*, tepals in 3 whorls, each with 3 or 4 tepals.	10. *Pilostyles thurberi*

### Allocation of all species names so far described in Apodanthaceae

#### 1. Apodanthes caseariae

Poiteau, Ann. Sci. Nat. (Paris) 3: 422, t. 26. 1824.

http://species-id.net/wiki/Apodanthes_caseariae

Apodanthes flacourtiae Karsten, Linnaea 28: 413. 1857. Type: Venezuela, Aragua, Choroni, parasitic on “Flacourtieae” [most like a species that today would be placed in Salicaceae], *H. Karsten s.n*. (W, destroyed in WWII), **syn. nov.**
Apodanthes tribracteata Rusby, Descr. S. Amer. Pl. 15. 1920. Type: Bolivia, near Inglis-Inglis, 8 Aug. 1902, *R. S. Williams 1580* (NY), **syn. nov.**
Apodanthes matogrossensis Vattimo, Vattimo-Gil, Rodriguésia 26(38): 45. 1971, without Latin descr. Type: Brazil, Mato Grosso, parasitic on *Casearia, J. G. Kuhlmann 53076* (R, not seen).**Nom. inval.**
Apodanthes panamensis Vattimo-Gil, Rodriguésia 26(38): 45. 1971, without Latin descr., Latin diagnosis in Rev. Brasil. Biol., 33(1): 140. 1973. Type: Panama, Canal Zone, Aug. 1984, *R. E. Woodson Jr. and R. W. Schery 965* (NY, MO). Already synonymized by A. Gentry (1973).
Apodanthes surinamensis Pulle, Recueil Trav. Bot. Néerl. 6: 259. 1909. Type: Suriname, along the Marowijne River, July-Dec. 1903, parasitic on Flacourtiaceae [most like a species that today would be placed in Salicaceae], *G. M. Versteeg s.n*. (U0007645), **syn. nov.**
Apodanthes roraimae Ida de Vattimo, Rodriguésia 29(44): 48-49. 1978. Type: Brazil, Roraima, 24 Jul. 1974, parasitic on Flacourtiaceae [most likely a species that today would be placed in Salicaceae], *G. T. Prance et al. 21353* (NY), **syn. nov.** Comment: George Yatskievych, a curator at the Missouri Botanical Garden, also studied the NY isotype in 2004 and annotated it as *Apodanthes caseariae*.
Apodanthes minarum Vattimo-Gil, Rodriguésia 26 (38): 45. 1971, without Latin descr.; Latin diagnosis in Rev. Brasil. Biol., 33(1): 140. 1973. Type: Brazil, Minas Gerais, Viçosa, 31 Mar. 1930, *Y. Mexia 4540* (L, MO), **syn. nov.**

##### Type.

French Guiana, Karouany, c. 1802, parasitic on *Casearia* spec., *P. A. Poiteau s.n*. (P: P00686413).

##### Note.

Tepals white to yellow, female flowers >5 mm long, tepals in 3 whorls, the outer with 2 tepals, the inner one easily deciduous (Figs 4A–D; 6C, D). Growing in trunk and branches of *Casearia* and occasionally *Xylosma* (Salicaceae, Fig. 3) in Guatemala, Honduras, Costa-Rica, Panama, Colombia, Venezuela, Suriname, French Guiana, Brazil, Peru and Bolivia (Fig. 2).

#### 2. Pilostyles collina

Dell, Nuytsia 4: 293–294. 1983.

http://species-id.net/wiki/Pilostyles_collina

##### Type.

Australia, Western Australia, Peak Charles, 10 Jan. 1982, parasitic on *Oxylobium*, *B. D. Dell 8216* (G, MO).

**Note:** Tepals orange to red, in 3 whorls. Growing in young stems of *Gastrolobium* and *Oxylobium* in Western Australia (Figs 2, 3, see Thiele et al. 2008 for pictures of flowers).

#### 3. Pilostyles hamiltonii

Gardner, J. Roy. Soc. Western Australia 32: 77. 1948.

http://species-id.net/wiki/Pilostyles_hamiltonii

##### Type.

Australia, Western Australia, Darling District, Helena Rover, Mundaring Weir, Mar. 1946, parasitic on *Daviesia pectinata* Lindl., *C. D. Hamilton s.n*. (PERTH, not seen).

**Note:** Tepals dark burgundy, in 2 whorls, flowers >3 mm in diameter. Growing in young stems of *Daviesia* in Western Australia (Figs 2, 3, see Thiele et al. 2008 for pictures of flowers).

#### 4. Pilostyles coccoidea

K.R.Thiele, Nuytsia 18: 273–284. 2008.

http://species-id.net/wiki/Pilostyles_coccoidea

##### Type.

Australia, Western Australia, Waddi Road, 30°33'26"S, 115°28'10"E, 7 Mar. 2008, parasitic on *Jacksonia*, *K.R. Thiele 3495* (PERTH 07692447).

##### Note.

Tepals pale orange to brown, in 2 whorls, flowers <3 mm in diameter. Growing in stems of *Jacksonia* in Western Australia (Figs 2 and 3, see Thiele et al. 2008 for pictures of flowers).

#### 5. Pilostyles aethiopica

Welwitsch, Trans. Linn. Soc. London 27: 66–70. 1871 = Berlinianche aethiopica (Welw.) Vattimo-Gil nom. inval.

http://species-id.net/wiki/Pilostyles_aethiopica

Pilostyles holtzii Engler, Bot. Jahrb. Syst. 46: 293. 1912 = *Berlinianche holtzii* (Engl.) Vattimo-Gil, not validly published. Type: Tanzania, Kilimatinde, July 1907, parasitic on *Berlinia eminii* Taub., *W. Holtz 1422* (B, destroyed during World War II), **syn. nov.** (based on the protologue).

##### Syntypes.

Angola, Huila, 12 May 1860, parasitic on *Berlinia paniculata* Benth. = *Pseudoberlinia paniculata* (Benth.) P.A.Duvign., *F. M. J. Welwitsch 529, 529b* (C, G).

##### Note.

Tepals pink to brown, in 3 to 4 whorls each with 3-6 tepals. Male flowers with 1 or 2 ring(s) of ca. 15 pollen sacs, stamen filaments free from the central column (Fig. 6F), hair cushion at the basis of the inner tepals (Blarer et al. 2004). Growing in branches of *Julbernardia* and *Brachystegia*, maybe also on *Berlinia*, *Westia* and *Pseudoberlinia*, in Zimbabwe, Zambia, Tanzania, Angola and Malawi (Figs 2, 3).

#### 6. Pilostyles haussknechtii

Boissier, Arch. Sci. Phys. Nat. 25: 255–261. 1866.

http://species-id.net/wiki/Pilostyles_haussknechtii

##### Type.

Middle East, parasitic on *Astragalus*, *J. E. Haussknecht s.n* (G-BOISS, not seen).

**Note:** Tepals pink to brown in 2 whorls, each with 6 to 10 tepals (Fig. 6G). Found at the basis of young branches of *Astragalus* and occasionally *Onobrychis* and *Halimodendron* in Iran (Figs 2, 3).

#### 7. Pilostyles berteroi

Guillemin, Ann. Sci. Nat., Bot., sér. 2, 2: 21. 1834 = Apodanthes berteroi (Guill.) Gardner, Hooker’s Icon. Pl. 7: t. 655. 1844. syn. nov.

http://species-id.net/wiki/Pilostyles_berteroi

##### Syntypes.

Chile, Quillota, parasitic on *Adesmia*, [in Chile 1828-1831] *C. L. G. Bertero s.n*. (P, not seen); Chile [from the collection number this was in “various localities in the Andes”, during the period from 27 Oct.-26 Nov. 1841], *T. Bridges 1273* (BM, not seen, K, not seen).

**Note:** Tepals purple to brown with clearer margins (Fig. 6A), 9–18 in number, stamens in 4 whorls (spirals), with > 70 pollen sacs. Growing in older stems of *Adesmia* shrubs in Chile, Argentina, Peru, and Bolivia (Figs 2, 3). Our morphological (Suppl. material 2) and molecular data (Fig. 1) show that the species is nested among other species of *Pilostyles*, indicating that Gardner’s transfer was erroneous.

#### 8. Pilostyles blanchetii

(Gardner) R.Br., Trans. Linn. Soc. London 19(3): 247. [6 Nov 1844] = Apodanthes blanchetii Gardner, Icon. Pl. 7: t. 655 b. 1844 [Jul 1844] = Frostia blanchetii (Gardner) H.Karst., Nov. Actorum Acad. Caes. Leop.-Carol. Nat. Cur. 26: 922. 1858.

http://species-id.net/wiki/Pilostyles_blanchetii

Pilostyles calliandrae (Gardner) R.Br., Trans. Linn. Soc. London 19(3): 247. [6 Nov 1844] = *Apodanthes calliandrae* Gardner, Icon. Pl. 7: t. 644. 1844 [Jan 1844] = *Frostia calliandrae* (Gardner) H. Karst., Nov. Actorum Acad. Caes. Leop.-Carol. Nat. Cur. 26: 921. 1858. Type: Brazil, Amazonas, near Maynas [Manaus], Feb. 1840, *G. Gardner 3639* (K000601222), **syn. nov.**
Pilostyles caulotreti (Karsten) Hook.f., Prodr. (DC.) 17: 116. 1873 *= Sarna caulotreti* Karsten, Linnaea 28: 415. Jun 1857 [1856]. Type: Venezuela, *H. Karsten s.n*. (W, destroyed in WWII). Comment: Gentry (1973) considered this name as synonym of *Pilostyles blanchetii*, and we agree with this assessment.
Pilostyles ingae (Karsten) Hooker f., Prodr. (DC.) 17: 116. 1873 = *Sarna ingae* H.Karst., Linnaea 28: 415. Jun 1857 [1856]. Type: Colombia, Cauca, Popayán, parasitic on *Inga, H. Karsten s.n*. (W, destroyed in WWII), **syn. nov.** (based on the protologue).
Pilostyles galactiae Ule, Notizbl. Königl. Bot. Gart. Berlin 6: 292. 1915. Type: Brazil, Amazonia, Surumu River, tributary of the Rio Branco, Oct. 1909 and Mar. 1910, parasitic on *Galactia jussiaeana* Kunth., *E. Ule 7895* (B, holotype destroyed in WWII; isotype NY), **syn. nov.**
Pilostyles goyazensis Ule, Ber. Deutsch. Bot. Ges. 33: 475. 1915. Syntypes (all parasitic on *Mimosa*): Brazil, Goias, region near city of Corumba, Sobradinho, Aug. 1892, *E. Ule 3097*; Serra dos Pyreneos, Mun. Corumba, Dec. 1892, *E. Ule 3098*; same location, Dec. 1892, *E. Ule 3099*; in the Corumba region, Aug. 1892, not flowering, *E. Ule s.n*.; Serra dos Pyreneos, Aug. 1892, not reproductive, *E. Ule s.n*. (all in B, material destroyed in WWII), **syn. nov.** (based on the protologue).
Pilostyles globosa (S.Watson ex Robinson) Hemsl., J. Linn. Soc., Bot. 31: 311. 1896 = *Apodanthes globosa* S.Watson ex Robinson., Bot. Gaz. 16: 83, tab. 9, 1891. Type: Mexico, Northern part, Sierra Madre, parasitic on *Bauhinia lunarioides* A. Gray, *C. G. Pringle 1950* (G), **syn. nov.**
Pilostyles stawiarskii Vattimo-Gil, Revista Brasil. Biol. 10: 196. 1950. Type: Brazil, Paraná, Mun. de Palmas, parasitic on *Mimosa scabrella* Benth. [incl. its synonym *Mimosa bracaatinga* Hoehne], Jan. 1948 and Feb. 1948, *V. Stawiarski* R50.591 and 50.592 (R, photos). There is also a topotypical collection from Dec. 1949, **syn. nov.** (based on the protologue).
Pilostyles ulei Solms-Laub., in Goebel, Organogr. Pfl. 2,1: 434. Figure 292 (1900), descr. in Endriss, Flora, Ergänz.-Bd. 91: 209. 1902. Type: Brazil, Goias, parasitic on Fabaceae, *E. Ule s.n*. (B, destroyed in WWII; R has E. Ule 34, E. Ule 36, E. Ule 38, E. Ule 148, E. Ule 367, E. Ule 482, and E. Ule 483 labeled as this species, not seen). Comment: already Solms-Laubach (1901) and Endriss (1902) considered *Pilostyles ulei* as a synonym of *Pilostyles ingae*.

##### Type.

Brazil: Bahia, 1839, *J. S. Blanchet 2861* (NY).

##### Note.

Tepals purple to brown sometimes with clearer margins (Fig. 6E), in 3 whorls with usually 4 (rarely 3-6) tepals, the middle tepal diamond-shaped. Stamens in 2 whorls. Found in branches of *Mimosa* and *Bauhinia*, but also *Cassia*, *Dioclea*, *Galactia* and *Schnella*, in Jamaica, Cayman Islands, Costa-Rica, Panama, Colombia, Venezuela, Guyana, Brazil, Argentina and Uruguay (Figs 2, 3).

#### 9. Pilostyles mexicana

(Brandegee) Rose, Contr. U.S. Natl. Herb. 12(7): 264. 1909 = Apodanthes mexicana Brandegee, Zoe 5(11): 245. 1908.

http://species-id.net/wiki/Pilostyles_mexicana

##### Type.

Mexico, near Zacuapan, Tenampa, parasite on *Calliandra grandiflora* Benth., Oct. 1906, *C.A. Purpus 2207* (NY).

##### Note.

Tepals red to brown, in 3 whorls, each with 4 tepals. Growing in branches of *Calliandra* in Guatemala, Honduras and Mexico (possibly further south; Figs 2, 3).

#### 10. Pilostyles thurberi

Gray, Pl. Nov. Thurb. 326–327. 1854.

http://species-id.net/wiki/Pilostyles_thurberi

Pilostyles covillei Rose, Contr. U.S. Natl. Herb. 12: 263. 1909. Type: USA, Texas, Dickens county, Matador ranch, 14 June 1894, parasitic on *Parosela formosa* (Torr.) Vail, *F. V. Coville 1860* (US, not seen).
Pilostyles glomerata Rose, Contr. U.S. Natl. Herb. 12: 263. 1909. Type: Mexico, Puebla, near Tehuacán, 31 Aug. 1905, parasitic on *Parosela*, *J. N. Rose and J. H. Painter 9942* (NY, G). The protologue gives the collection number as 8942.
Pilostyles palmeri Rose, Contr. U.S. Natl. Herb. 12: 263. 1909. Type: Mexico, San Luis Potosí, near Alvarez, May 1887, parasitic on *Parosela*, *E. Palmer 584* (US-570088).
Pilostyles pringlei (Watson) Hemsl., J. Linn. Soc., Bot. 31: 311. 1896 = *Apodanthes pringlei* Watson ex B.L.Rob., Bot. Gaz. 16: 83, tab. 9. 1891, no Latin descr.; *Pilostyles pringlei* (Watson) Rose, Contr. U.S. Natl. Herb. 12: 264. 1909, superfluous transfer. Type: Mexico, Sierra Madre, near Monterey, 27 June 1888, parasitic on *Dalea frutescens* A. Gray, *C. G. Pringle 1949* (NY, M, G).
Pilostyles sessilis Rose, Contr. U.S. Natl. Herb. 12: 263. 1909. Type: male flowers: Mexico, Hidalgo, Ixmiquilpan, 1905, parasitic on *Parosela*, *J. N. Rose 9041* (NY); female flowers: Mexico, Querétaro, hacienda Ciervo, 20 Aug. 1905, parasitic on *Parosela tuberculata* (Lag.) Rose, *J. N. Rose and J. H. Painter 9636* (NY, US).

##### Type.

USA, probably Arizona, near Gila river, June 1850, parasitic on *Psorothamnus emoryi* (A. Gray) Rydb., *G. Thurber 682* (NY).

##### Note.

Tepals white, red to brown, in 3 whorls, each with 3 or 4 tepals, rarely more (Fig. 6B). Growing in branches of *Dalea*, *Parosela* and *Psorothamnus* in the southern United States of America and Mexico (Figs 2, 3). New York (NY) has a specimen from Mexico of this species annotated as “*Pilostyles mortoni*”, a nomen nudum, by Ida de Vattimo in 1952.

### Note on an invalid genus name

Harms (1935) tried to place the two African names, *Pilostyles aethiopica* Welw. and *Pilostyles holtzii* Engl., in a separate section, *Pilostyles* section *Berlinianche*, named for their legume host species in the genus *Berlinia*, but failed to include a Latin diagnosis for the new section. Later, Vattimo-Gil (1955, 1971) decided to rank this section as a separate genus because of the hair cushions on the inner perianth whorl and strictly tri- and hexamerous flowers compared to the tetramerous flowers of the American species of *Pilostyles*. This assessment, however, could only have been based on specimens of *Pilostyles aethiopica*, since the only collection of *Pilostyles holtzii* burnt in World War II. Unfortunately, Vattimo-Gil also neglected to provide a Latin diagnosis, and the genus name is therefore not valid. Based on our results (Fig. 1), *Pilostyles aethiopica* does not deserve generic status because it is embedded among the other species of *Pilostyles*.

### Note on a possible new species of *Pilostyles*

Flavio González and Natalia Pabón-Mora, at the university of Antioquia in Colombia, are studying the ecology and morphology of Apodanthaceae in Colombia (González and Pabón-Mora accepted a) and are describing a new species of *Pilostyles* (González and Pabón-Mora accepted b). This species is the first *Pilostyles* parasitizing the legume genus *Dalea* in South America and occurs in dry valleys of the Colombian Eastern Cordillera at altitudes above 2000 m. Morphologically, the new species is most similar to *Pilostyles berteroi*, which grows in the Chilean and Peruvian Andes at up to 3000 m of altitude (Fig. 2) and parasitizes *Adesmia* (closely related to *Dalea*, see Fig. 3).

## Conclusion

By combining morphological and molecular information, we show that Apodanthaceae comprise 10 species and that morphological distinctions fit well with geographical disjunctions and specializations on different hosts (Salicaceae vs. Fabaceae). DNA sequences of mitochondrial *matR* and nuclear 18S rDNA, along with morphology and geography permit identifying any collection of Apodanthaceae. A wider sampling of the morphological variation, especially of male *Apodanthes caseariae* and female *Pilostyles blanchetii*, *Pilostyles mexicana* and *Pilostyles thurberi*, however, is needed to determine whether some unusual specimens might deserve to be ranked as subspecies.

## Supplementary Material

XML Treatment for Apodanthes caseariae

XML Treatment for Pilostyles collina

XML Treatment for Pilostyles hamiltonii

XML Treatment for Pilostyles coccoidea

XML Treatment for Pilostyles aethiopica

XML Treatment for Pilostyles haussknechtii

XML Treatment for Pilostyles berteroi

XML Treatment for Pilostyles blanchetii

XML Treatment for Pilostyles mexicana

XML Treatment for Pilostyles thurberi

## Supplementary materials

Supplementary material 1 List of accessions used in this study with author names for each species, herbarium vouchers, and GenBank accession numbers doi: 10.3897/phytokeys.36.7385.app1 Data type: species list This dataset is made available under the Open Database License (http://opendatacommons.org/licenses/odbl/1.0/). The Open Database License (ODbL) is a license agreement intended to allow users to freely share, modify, and use this Dataset while maintaining this same freedom for others, provided that the original source and author(s) are credited.Sidonie Bellot, Susanne S. Renner

Supplementary material 2 Results of the dissections of 123 flowers from 82 specimens of Apodanthaceae doi: 10.3897/phytokeys.36.7385.app2 Data type: measurements This dataset is made available under the Open Database License (http://opendatacommons.org/licenses/odbl/1.0/). The Open Database License (ODbL) is a license agreement intended to allow users to freely share, modify, and use this Dataset while maintaining this same freedom for others, provided that the original source and author(s) are credited.Sidonie Bellot, Susanne S. Renner
