# Supplementary material for: The systematics of the worldwide endoparasite family Apodanthaceae (Cucurbitales), with a key, a map, and color photos of most species
Source: PhytoKeys. 2014 Apr 30;(36):41–57. doi: 10.3897/phytokeys.36.7385 (PMC4023342; doi:10.3897/phytokeys.36.7385)
Supplement: Supplementary material 1 — List of accessions used in this study with author names for each species, herbarium vouchers, and GenBank accession numbers [file phytokeys-036-041-s001.doc]

| Species name | Voucher (Herbarium)  18S / matR | Source  18S / matR | GenBank accession  18S | GenBank accession *matR* |
| --- | --- | --- | --- | --- |
| *Apodanthes caseariae* Poiteau | R. Callejas 8062 (NY) | This paper | KJ634137 | KJ634133 |
| *Apodanthes caseariae* Poiteau | C. Galdames 7260 (M) | Bellot and Renner, in review | KJ634128 | KJ634100 |
| *Apodanthes caseariae* Poiteau | Y. Mexia 4540 (MO) | This paper | KJ634138 | KJ634134 |
| *Apodanthes caseariae* Poiteau | D. N. Smith et al. 14057 (MO) | Bellot and Renner, in review | KJ634127 | KJ634101 |
| Probably *Casearia sp.* | Host bark of R. Callejas 8062 (NY) | This paper | - | KJ634135 |
| *Corynocarpus laevigatus* J.R.Forst & G.Forst | ^1^M. W. Chase 236 (NCU); ^2^M. W. Chase s.n. (NCU) | ^1^Soltis et al., 2000; ^2^Zhu et al., 2007 | AF206892^1^ | AY121499^2^ |
| *Pilostyles aethiopica* Welwitsch | S. Bellot 29 (M) | Bellot and Renner, in review | KJ634129 | - |
| *Pilostyles berteroi* Guill. | J. N. Rose and L. B. Rose 18849 (NY) | This paper | - | KJ634136 |
| *Pilostyles berteroi* Guill. | C. Heibl 05-017 (M) | Bellot and Renner, in review | KJ634126 | KJ634102 |
| *Pilostyles blanchetii* (Gardner) Brown | H. S. Irwin et al. 20350 (NY) | This paper | - | KJ634132 |
| *Pilostyles blanchetii* (Gardner) Brown | E. P. Heringer et al. 5768 (MO) | Bellot and Renner, in review | KJ634125 | KJ634103 |
| *Pilostyles blanchetii* (Gardner) Brown | H. S. Irwin et al. 31560 (MO) | This paper | KJ634139 | - |
| *Pilostyles blanchetii* (Gardner) Brown | H. S. Irwin et al. 14880 (MO) | Bellot and Renner, in review | KJ634122 | KJ634106 |
| *Pilostyles blanchetii* (Gardner) Brown | A. Glaziou 22029 (G) | Bellot and Renner, in review | KJ634119 | KJ634108 |
| *Pilostyles blanchetii* (Gardner) Brown | G. T. Ceccantini et al. 3593 (SPF) | Bellot and Renner, in review | KJ634114 | KJ634113 |
| *Pilostyles blanchetii* (Gardner) Brown | M. Nee et al. 48695 (NY) | Bellot and Renner, in review | KJ634115 | KJ634112 |
| *Pilostyles coccoidea* K.R.Thiele | K. R. Thiele 3495 (PERTH) | Bellot and Renner, in review | KJ634124 | KJ634104 |
| *Pilostyles collina* B.Dell | K. R. Thiele 4501 (PERTH) | Bellot and Renner, in review | KJ634123 | KJ634105 |
| *Pilostyles hamiltonii* C.A.Gardner | D. Dixon 1039 (PERTH) | Bellot and Renner, in review | KJ634121 | KJ634107 |
| *Pilostyles haussknechtii* Boissier | A. Chehregani and S. Zarre 17834 (M) | Bellot and Renner, in review | KJ634120 | - |
| *Pilostyles mexicana* (Brandegee) Rose | D. E. Breedlove 27233 (NY) | Bellot and Renner, in review | KJ634118 | KJ634109 |
| *Pilostyles thurberi* Gray | F. Lyle Wynd and C. H. Mueller 256 (NY) | Bellot and Renner, in review | KJ634117 | KJ634110 |
| *Pilostyles thurberi* Gray | F. Chiang 9034 (NY) | This paper | - | KJ634131 |
| *Pilostyles thurberi* Gray | M. C. Johnston et al. 11286 (MO) | Bellot and Renner, in review | KJ634116 | KJ634111 |
| *Pilostyles thurberi* Gray | D. L. Nickrent 4342 (SIU) | Nickrent et al., 2004 | - | AY739003 |
| *Pilostyles thurberi* Gray | J. Rzedowski 11303 (A) | This paper | - | KJ634130 |

Nickrent, D.L., Blarer, A., Qiu, Y.L., Vidal-Russell, R., Anderson, F.E., 2004. Phylogenetic inference in Rafflesiales: the influence of rate heterogeneity and horizontal gene transfer. BMC Evol. Biol. 4, 40.

Soltis, D.E., Soltis, P.S., Chase, M.W., Mort, M.E., Albach, D.C., Zanis, M., Savolainen, V., Hahn, W.H., Hoot, S.B., Fay, M.F., Axtell, M., Swensen, S.M., Prince, L.M., Kress, W.J., Nixon, K.C., Farris, J.S., 2000. Angiosperm phylogeny inferred from 18S rDNA, *rbcL*, and *atpB* sequences. Botanical Journal of the Linnean Society 133, 381–461.

Zhu, X.-Y., Chase, M.W., Qiu, Y.-L., Kong, H.-Z., Dilcher, D.L., Li, J.-H., Chen, Z.-D., 2007. Mitochondrial matR sequences help to resolve deep phylogenetic relationships in rosids. BMC Evolutionary Biology 7, 217.
